# Rapid evaluation for health and social care innovations: challenges for “quick wins” using interrupted time series

**DOI:** 10.1186/s12913-019-4821-7

**Published:** 2019-12-13

**Authors:** Andrew McCarthy, Peter McMeekin, Shona Haining, Lesley Bainbridge, Claire Laing, Joanne Gray

**Affiliations:** 1 Department of Health and Life Sciences, Northumbria University, Coach Lane Campus West, Newcastle Upon Tyne, NE77XA UK; 2 Institute of Health and Wellbeing, Glasgow University, 1 Lily bank Gardens, Glasgow, G12 8RZ UK; 3 Head of Research & Evidence, North of England Commissioning Support (NECS), Riverside House, Goldcrest Way, Newburn Riverside, Newcastle, NE15 8NY UK; 4 NHS Newcastle Gateshead Clinical Commissioning Group, Riverside House, Goldcrest Way, Newcastle upon Tyne, NE1 8NY UK; 5 Business Intelligence, North of England Commissioning Support, John Snow House, Durham, DH1 3YG UK

**Keywords:** Interrupted time series, Complex interventions, Health and social care, Vanguards, Service evaluation

## Abstract

**Background:**

Rapid evaluation was at the heart of National Health Service England’s evaluation strategy of the new models of care vanguard programme. This was to facilitate the scale and spread of successful models of care throughout the health & social care system. The aim of this paper is to compare the findings of the two evaluations of the Enhanced health in Care Homes (EHCH) vanguard in Gateshead, one using a smaller data set for rapidity and one using a larger longitudinal data set and to investigate the implications of the use of rapid evaluations using interrupted time series (ITS) methods.

**Methods:**

A quasi-experimental design study in the form of an ITS was used to evaluate the impact of the vanguard on secondary care use. Two different models are presented differing by timeframes only. The short-term model consisted of data for 11 months data pre and 20 months post vanguard. The long-term model consisted of data for 23 months pre and 34 months post vanguard.

**Results:**

The cost consequences, including the cost of running the EHCH vanguard, were estimated using both a single tariff non-elective admissions methodology and a tariff per bed day methodology. The short-term model estimated a monthly cost increase of £73,408 using a single tariff methodology. When using a tariff per bed day, there was an estimated monthly cost increase of £14,315.

The long-term model had, using a single tariff for non-elective admissions, an overall cost increase of £7576 per month. However, when using a tariff per bed-days, there was an estimated monthly cost reduction of £57,168.

**Conclusions:**

Although it is acknowledged that there is often a need for rapid evaluations in order to identify “quick wins” and to expedite learning within health and social care systems, we conclude that this may not be appropriate for quasi-experimental designs estimating effect using ITS for complex interventions. Our analyses suggests that care must be taken when conducting and interpreting the results of short-term evaluations using ITS methods, as they may produce misleading results and may lead to a misallocation of resources.

## Background

The NHS 5 Year Forward View [1] set out the strategic plan for the NHS and included within it a number of challenges to the models of care required to meet changing patient and carer needs. It established 50 vanguard sites to take the lead on 5 new models of care with a key facet being improved integration within the system of care delivery to improve outcomes, and ensuring care and treatment were delivered in the most appropriate and efficient setting. One of the models identified was the Enhanced Health in Care Homes (EHCH). Against the backdrop of the need to break down barriers in care provision, 1 in 7 people over 85 living in a long term care setting, and spending increasingly significantly with age, 6 EHCH vanguard sites were set up nationally to challenge ways of working and improve integration and outcomes. Key outcomes include helping frail and older people to stay healthy and independent and reduce unnecessary hospital admissions, and reviewing models of working and contracting arrangements [2].

The Gateshead Enhanced Health in Care Homes new model of care was selected to be one of these vanguards and was launched in March 2015. The vanguard’s purpose was to increase collaborative working and establish partnerships between health and care providers to improve the health and wellbeing of residents and thereby reduce pressure on primary, secondary and social care services. The vanguard consisted of three key features: Link GP Practices, Older Person Specialist Nurses (OPSN), and Multi-Disciplinary Teams (MDTs). The link GP Practices strand of the vanguard consists of signing up residents of a care home to the same GP practice (usually the closest geographically). Older Person Specialist Nurses were also assigned to care homes in both localities in order to support care home staff in delivering care. Furthermore, each care home had a Multi-Disciplinary Team (MD) which consisted of key healthcare professionals such as; geriatric consultants, link GPs, and specialist nurses.

Evaluation of these vanguards was at the heart of the programme: enabling the widespread adoption of new models of care that improve the health and wellbeing of patients; the quality and equality of care that patients receive; and the efficiency of the overall system [3]. Indeed, there was an emphasis as part of the evaluation strategy that findings should be shared rapidly among the vanguards and spread throughout the NHS. This echoes a relatively recent phenomenon of a rapid cycle of evaluation in health services research.

Rapid evaluation as a strategy for evaluation gained traction in 2018 with the creation of two National Institute for Health Research (NIHR) funded research centres, the Birmingham RAND and Cambridge Evaluation (BRACE), and the Rapid Service Evaluation Team (‘RSET’) these were created to conduct rapid evaluations of promising new services and innovations in healthcare over a five-year programme.

A local evaluation for the Gateshead EHCH vanguard was commissioned to quantitatively evaluate the impact of the vanguard in terms of effect and value for money. This evaluation was conducted during the first 2 years of the roll out of the vanguard and was part of the evaluation strategy for rapid evaluation and learning across the health and social care economy. In addition to this initial evaluation, a further evaluation was conducted utilising a much larger data set with more pre and post intervention data points. The aim of this paper is to present and compare the findings of the two evaluations and to investigate the implications of the use of rapid evaluations using interrupted time series (ITS) methods.

## Methods

### Design

A quasi-experimental design study in the form of an ITS was used to evaluate the impact of the EHCH in Gateshead As all the nursing and residential homes were covered by the vanguard which was introduced in March 2015. Two different models are presented which differ due to the timeframes only. The short-term model consisted of data from April 2014 to October 2016, resulting in 11 months before the introduction of the ECHC vanguard and 20 months afterwards. The long-term model consisted of data from April 2013 to December 2017, resulting in 23 months before the introduction of the EHCH vanguard and 34 months afterwards.

Gateshead contains 34 of residential and nursing care homes (excluding learning disability care homes) with 1503 beds. Data was obtained from the North of England Commissioning Support Unit (NECS) for monthly Secondary Users Service (SUS) data for total numbers of Accident & Emergency (A&E) attendances, non-elective admissions (excluding ambulatory care), outpatient appointments and bed-days in secondary care use. Due to the absence of an indicator within the SUS dataset for care home residents, a proxy was used to identify the care home population. As most care home residents in Gateshead are aged over 80, It was assumed that an individual with an age of 80 of higher who lived within the post-code area of the care homes in Gateshead was a care home resident.

### Statistical analysis

The impact of the introduction of the vanguard on secondary care use was assessed using a log linear ITS Ordinary Least Squares (OLS) regression. The two models are presented below.

Short-term model:

$$ \ln \left({Y}_t\right)={\beta}_1+{\beta}_2{T}_t+{\beta}_3W+{\beta}_4D+{\beta}_5\left(T-{T}_{11}\right) $$

Long-term model:

$$ \ln \left({Y}_t\right)={\beta}_1+{\beta}_2{T}_t+{\beta}_3W+{\beta}_4D+{\beta}_5\left(T-{T}_{23}\right) $$

Where ln (Y_t_) shows the proportional change in each of the outcomes. T_t_ is the underlying time trend before the introduction of the EHCH vanguard of care. *β*_2_ is a co-efficient that shows the percentage change in the relevant outcome for each month prior to the EHCH. W is a dummy variable for winter months (this variable takes a value of one from November to February and zero otherwise). *β*_3_ is the coefficient for the winter variable that shows the estimated percentage change in the relevant outcome as a consequence of the impact of winter. D is the dummy variable showing the impact on outcome for the period immediately following the vanguard (it takes a value of zero before the start of the EHCH and a value of one after the start of the EHCH in March 2015). *β*_4_ is the coefficient of D which shows a percentage change in the outcome in the period immediately after the introduction of the vanguard. (*T* − *T*_*x*_) is an interaction term which shows the change in the time trend following the introduction of the vanguard. This differs between the short-term model and long-term model due to the different lengths of the pre-intervention time-periods in each model, showing the percentage step-change of the outcome following the introduction of the vanguard. *β*_5_ is the coefficient which shows the percentage change of the time trend following the introduction of the vanguard.

Results of the analyses are reported in tables with the coefficient value (standard error), 95% confidence interval, and significance at the 95% level reported through the *p*-value. Each model was evaluated in terms of the underpinning assumptions of OLS to ensure best linear unbiased estimates were obtained. The cumulative probability plots of residuals (PP plots) were used to assess the normality of the residuals and are reported in Additional file 1. Heteroscedasticity was assessed though the Breusch-Pagan/Cook Weisberg test [4]. Autocorrelation was investigated using the Breusch-Godfrey test [5, 6] to allow investigation beyond first order autocorrelation. Where autocorrelation was identified, the Newey-West [7] method for correcting OLS standard errors was used. Results of the tests for heteroscedasticity and Autocorrelation are reported in Additional file 2.

For each analysis, a graph is presented which plots the resource use against time. Reported are the data points (dots), the predicted values of the model (black line) and the alternative predicted model had the EHCH vanguard not occurred (dashed line).

#### Methods for health economics

In order to estimate the cost-consequences of the EHCH intervention, the cost of the predicted resource use from the regressions for each outcome were compared to the counterfactual, that is the predicted value assuming only the time and winter effects had occurred (i.e. what would have happened assuming the pre-EHCH trends continued over the length of the model). Although total cost implications of the time periods of each model is reported, comparisons will be of the average monthly cost implications due to the different follow-up times between the short-term and long-term models.

The cost of running the enhanced health in care homes vanguard was calculated using micro costing from an NHS perspective with each component cost shown in Table 1. The calculation of the cost of the care model assumed that an MDT meeting took place for 4 hours for 52 weeks of the year. Furthermore, it was assumed that two care plans took place annually per care-home bed.

**Table 1:** Component costs of the enhanced care in care homes vanguard

Resource Use	Cost (£)	Total cost (£)	Source
GP link cost	4000 per care home	120,000	Newcastle-Gateshead CCG
Practice educator	36,250 per year	36,250	Mid-point grade 7 agenda for change pay scale (2016) [8]
Dietician	30,375 per year	30,375	Mid-point grade 6 agenda for change pay scale [8]
6 Older-Person Specialist Nurses	36,250 per nurse per year	217,500	Mid-point grade 7 agenda for change pay scale [8]
Old-age Psychiatrist	138 per hour	28,704^a^	PSSRU [9]
2 Geriatric Consultants	135 per hour per consultant	56,160 ^a^	PSSRU [9]
1503 care home beds receiving care plans	£100 per care plan	300,600 ^b^	Newcastle-Gateshead CCG

^a^ Assumed the MDT meeting is 4 h long and takes place 52 weeks per year

^b^ Assumed two care plans per care home bed every year

The unit costs used for this analysis are included in Table 2. With regards to the costs of non-elective admissions, two different methods were used; firstly a single tariff for each non-elective admission irrespective of length of stay, and secondly a tariff per bed day. As such, the overall cost implications are reported according to non-elective admissions or bed days perspectives as the sum of the resources use and the cost of providing the EHCH vanguard.

**Table 2:** Unit cost for resource use

Resource Use	Unit Cost (£)	Source
A&E attendance	138	NHS reference costs 2015/16 [10]
Non-elective admission	3058	NHS reference costs 2015/16 [10]
Outpatient appointments	227	NHS reference costs 2015/16 [10]
Non-elective bed day (enhanced tariff option)	222	NHS reference costs 2015/16 [10]

## Results

### A&E attendances

The results of the ITS regressions for A&E attendances of the two models are presented in Table 3. For the short-term model, there was an estimated pre-EHCH decrease in A&E attendances by 0.2% (*p* = 0.915) monthly. There was an additional winter effect estimated as an increase of 11.4% (*p* = 0.153). Following the introduction of the EHCH vanguard, there was an initial step increase in A&E attendances of 7.1% (*p* = 0.587). The EHCH vanguard resulted in an estimated increase in the time trend of 1.2% (*p* = 0.509), resulting in a post-EHCH monthly increase in A&E attendances of 1%.

**Table 3:** Results of short and long-term models for A&E attendances

Model	Variables	Coefficients (SE)	Significance	Confidence Interval
Short-term model	Constant	4.264 (0.104)	< 0.001	4.050, 4.479
	Time	−0.002 (0.018)	0.915	−0.038, 0.034
	Winter DV	0.114 (0.077)	0.153	−0.045, 0.272
	Step Change DV	0.071 (0.128)	0.587	−0.193, 0.334
	Slope Change	0.012 (0.019)	0.509	−0.026, 0.051
Long-term model	Constant	4.028 (0.060)	< 0.001	3.906, 4.149
	Time	0.013 (0.005)	0.007	0.004, 0.022
	Winter DV	0.096 (0.042)	0.025	0.012, 0.180
	Step Change DV	0.076 (0.078)	0.333	−0.080, 0.232
	Slope Change	−0.011 (0.005)	0.030	−0.022, − 0.001

**Fig. 1:**
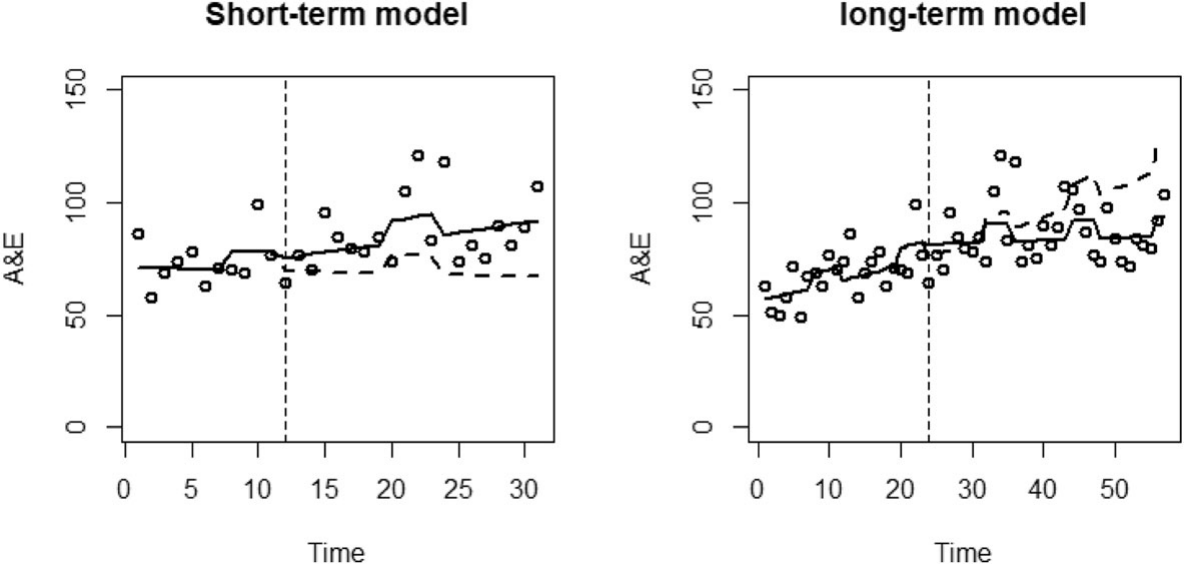
ITS regression results for A&E attendances of the short-term and long-term models

The long-term model estimates a monthly increase of 1.3% (*p* = 0.007) before the introduction of the EHCH, with an additional winter increase of 9.6% (*p* = 0.025). There was an estimated immediate increase in A&E attendances of 7.6% (*p* = 0.333) when the EHCH vanguard was started. The EHCH also resulted in an estimated reduction of 1.1% (*p* = 0.030) to the time trend, resulting in a post-EHCH monthly increase of 0.2% Fig. 1.

### Non-elective admissions

The results of the ITS regressions for non-elective attendances of the two models are presented in Table 4. For the short-term model, there was an estimated pre-EHCH decrease in non-elective admissions by 0.4% (*p* = 0.811) monthly. There was an additional winter effect estimated as an increase of 10.1% (*p* = 0.172). Following the introduction of the EHCH vanguard, there was an initial step reduction in non-elective attendances 20.7% (*p* = 0.096). The EHCH vanguard resulted in an estimated increase in the time trend of 0.9% (*p* = 0.606), resulting in a post-EHCH monthly increase in non-elective admissions of 0.5%.

**Table 4:** Results of short and long-term models for non-elective admissions

Model	Variables	Coefficients (SE)	Significance	Confidence interval
Short-term model	Constant	4.360 (0.097)	< 0.001	4.160, 4.560
	Time	− 0.004 (0.016)	0.811	−0.038, 0.030
	Winter DV	0.101 (0.072)	0.172	−0.047, 0.250
	Step Change DV	−0.207 (0.120)	0.096	−0.453, 0.039
	Slope Change	0.009 (0.017)	0.606	−0.027, 0.045
Long-term model	Constant	4.190 (0.064)	< 0.001	4.062, 4.318
	Time	0.008 (0.005)	0.106	−0.002, 0.018
	Winter DV	0.096 (0.044)	0.033	0.008, 0.184
	Step Change DV	−0.190 (0.082)	0.025	−0.354, − 0.025
	Slope Change	−0.011 (0.005)	0.041	−0.022, − 0.0005

**Fig. 2:**
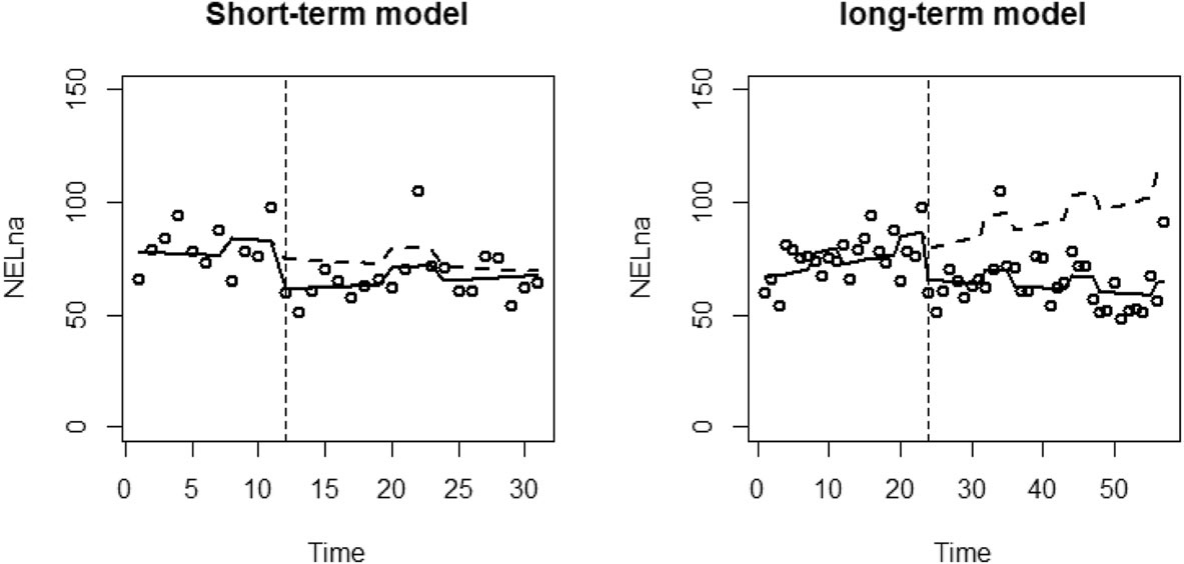
ITS regression results for non-elective attendances of the short-term and long-term models

The long-term model estimates a monthly increase of 0.8% (*p* = 0.106) before the introduction of the EHCH, with an additional winter increase of 9.6% (*p* = 0.033). There was an estimated immediate reduction in non-elective admissions of 19.0% (*p* = 0.025) when the EHCH vanguard was started. The EHCH also resulted in an estimated reduction of 1.1% (*p* = 0.041) to the time trend, resulting in a post-EHCH monthly reduction of 0.3% Fig. 2.

### Outpatient appointments

The results of the ITS regressions for outpatient appointments of the two models are presented in Table 5. For the short-term model, there was an estimated pre-EHCH decrease in outpatient appointments by 0.2% (*p* = 0.321) monthly. There was an additional winter effect estimated as an increase of 5.2% (*p* = 0.070). Following the introduction of the EHCH vanguard, there was an initial increase in outpatient appointments of 27.7% (*p* = 0.559). The EHCH vanguard resulted in an estimated increase in the time trend of 3.6% (*p* = 0.107), resulting in a post-EHCH monthly increase in outpatient appointments of 1.6%.

**Table 5:** Results of short and long-term models for outpatient appointments

Model	Variables	Coefficients (SE)	Significance	Confidence Interval
Short-term model	Constant	5.368 (0.119)	< 0.001	5.124, 5.613
	Time	−0.020 (0.020)	0.321	−0.062, 0.021
	Winter DV	0.052 (0.088)	0.070	−0.025, 0.578
	Step Change DV	0.277 (0.147)	0.559	−0.129, 0.234
	Slope Change	0.036 (0.021)	0.107	−0.008, 0.079
Long-term model	Constant	5.762 (0.043)	< 0.001	5.677, 5.847
	Time	−0.027 (0.004)	< 0.001	−0.035, − 0.019
	Winter DV	−0.025 (0.042)	0.562	−0.109, 0.060
	Step Change DV	0.371 (0.102)	0.001	0.166, 0.576
	Slope Change	0.032 (0.007)	< 0.001	0.019, 0.045

**Fig. 3:**
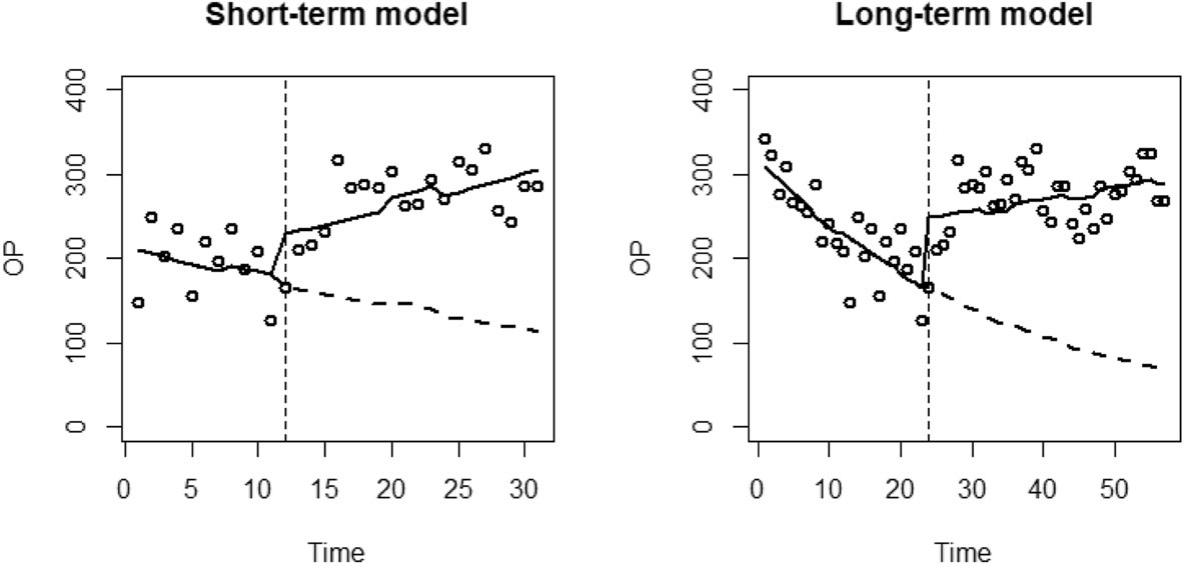
ITS regression results for outpatient appointments of the short-term and long-term models

Outpatient’s appointments for the long-term model showed third order autocorrelation. As such the Newey West model was implemented to account for this. The long-term model estimates a monthly reduction of 2.7% (*p* < 0.001) before the introduction of the EHCH, with an additional winter reduction of 2.5% (*p* = 0.562). There was an estimated immediate increase in outpatient appointments of 37.1% (*p* < 0.001) when the EHCH vanguard was started. The EHCH also resulted in an estimated increase of 3.2% (*p* < 0.001) to the time trend, resulting in a post-EHCH monthly increase of 0.5% Fig. 3.

### Bed-days

The results of the ITS regressions for bed days of the two models are presented in Table 6. For the Short-term model, there was an estimated pre-EHCH increase in bed days by 0.6% (*p* = 0.856) monthly. There was an additional winter effect estimated as an increase of 18.9% (*p* = 0.169). Following the introduction of the EHCH vanguard, there was an initial reduction in bed days of 40.5% (*p* = 0.079). The EHCH vanguard resulted in an estimated reduction of the time trend of 1.3% (*p* = 0.690), resulting in a post-EHCH monthly increase in non-elective admissions of 1.6%.

**Table 6:** Results of short and long-term models for bed days

Model	Variables	Coefficients (SE)	Significance	Confidence Interval
Short-term model	Constant	6.720 (0.180)	< 0.001	6.350, 7.089
	Time	0.006 (0.030)	0.856	− 0.057, 0.068
	Winter DV	0.189 (0.133)	0.169	−0.086, 0.463
	Step Change DV	−0.405 (0.221)	0.079	−0.860, 0.050
	Slope Change	−0.013 (0.032)	0.690	−0.079, 0.053
Long-term model	Constant	6.513 (0.103)	< 0.001	6.305, 6.720
	Time	0.014 (0.008)	0.089	−0.002, 0.029
	Winter DV	0.190 (0.071)	0.010	0.047, 0.333
	Step Change DV	−0.425 (0.133)	0.002	−0.692, − 0.158
	Slope Change	−0.023 (0.009)	0.010	−0.042, − 0.006

**Fig. 4:**
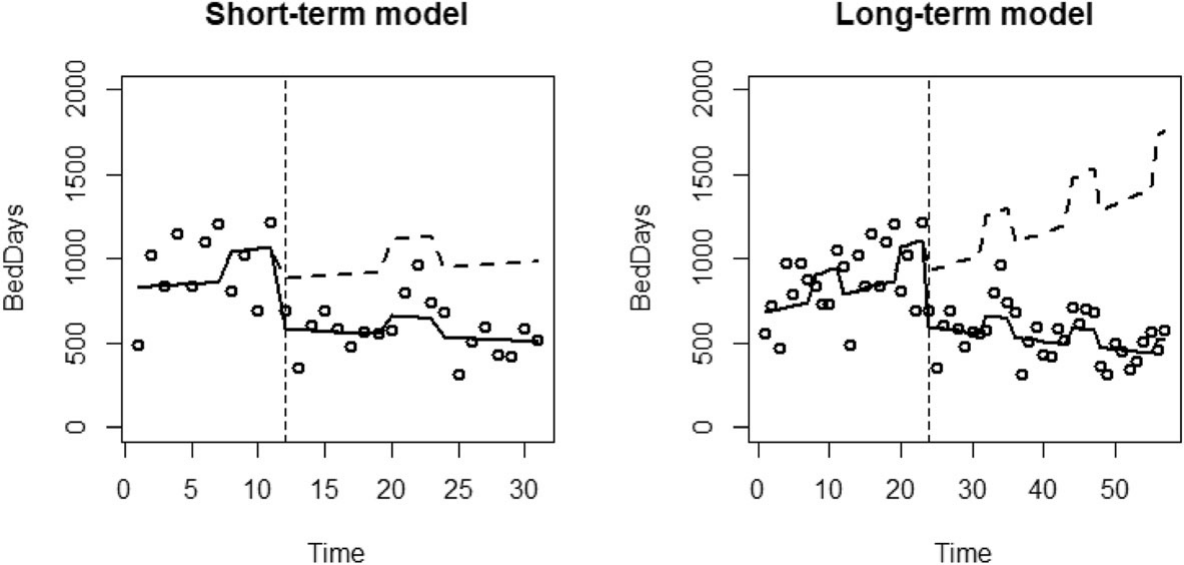
ITS regression results for A&E Bed days of the short-term and long-term models

The long-term model estimates a monthly increase of 1.4% (*p* < 0.089) before the introduction of the EHCH, with an additional winter increase of 19.0% (*p* = 0.0.10). There was an estimated immediate reduction in bed days of 42.5% (*p* = 0.002) when the EHCH vanguard was started. The EHCH vanguard also resulted in an estimated reduction of 2.3% (*p* = 0.010) to the time trend, resulting in a post-EHCH monthly increase of 0.9% Fig. 4.

### Health economic analysis

Presented in Table 7 is the total and average monthly cost implications for both the cost of running the EHCH vanguard and each resource use following the introduction of the EHCH vanguard; A&E attendance, non-elective admissions, outpatient appointments, and Bed-days for both the short-term model and long-term model. There was an estimated annual cost of £789,589 for providing the EHCH vanguard in Gateshead, producing an average monthly cost of £65,799. For the short-term model, the total cost of the model of care between March 2015 and October 2016 was £1,315,980. For the long-term model, the cost of the EHCH vanguard between March 2015 and December 2017 was £2,237,166. The short-term model reported an increased monthly cost of £2176 for A&E attendances and an increased monthly cost of £29,075 for outpatient appointments as a consequence of the introduction of the EHCH vanguard. Additionally, a monthly cost saving was estimated for non-elective admissions based on a single tariff of £23,641 and a monthly cost saving for bed days of £90,344. The longer-term model had an estimated average monthly cost increase only for outpatient appointments of £36,429. There was an estimated monthly cost saving in the long-term model for A&E attendances of 1670, for single tariff non-elective admissions of £92,982, and for bed-days of £177,726.

**Table 7:** Cost implications of resource use based on 2015/2016 costs

Resource	Cost measure	Short-term model	Long-term model
Cost of running ECHC vanguard	Total cost (£)	1,315,980	2,237,166
	Monthly cost (£)	65,799	65,799
A&E attendances	Total cost (£)	43,524	−56,781
	Monthly cost (£)	2176	− 1670
Non-elective admissions	Total cost (£)	− 472,828	−3,161,391
	Monthly cost (£)	−23,641	−92,982
Outpatient appointments	Total cost (£)	581,490	1,238,578
	Monthly cost (£)	29,075	36,429
Bed-days	Total cost (£)	−1,806,871	−5,362,668
	Monthly cost (£)	−90,344	− 177,726

Table 8 presents the estimated cost consequences of the EHCH vanguard with regards to both a single tariff non-elective admissions methodology and a tariff per bed day methodology. For the short-term model, there was as estimated monthly increase of £7610 as a consequence of the changes in non-elective admissions, outpatient appointments and A&E attendances. With a monthly cost of the ECHC vanguard of £65,799, the short-term model estimated a monthly cost increase of £73,408 using a single tariff methodology. However, when using a tariff per bed day, there was an estimated reduction in costs of £59,093 resulting in a monthly cost increase of £14,315 when taking into account the cost of running the EHCH vanguard.

**Table 8:** Overall cost implications

Cost implications	Cost Measure	Short-term model	Long-term model
Single tariff non-elective admissions perspective	Total cost (£)	1,468,166	257,572
	Monthly cost (£)	73,408	7576
Tariff per bed day perspective	Total cost (£)	286,308	−1,943,705
	Monthly cost (£)	14,315	−57,168

The long-term model had, using a single tariff for non-elective admissions, an estimated average monthly reduction of £58,223 as consequence resource use. When accounting for the cost of the EHCH vanguard, there was an overall cost increase of £7576 per month. However, when using a tariff per bed-days, there was an estimated monthly cost reduction in resource use of £122,967. When taking into account the cost of the EHCH vanguard, this resulted in an estimated cost reduction of £57,168.

## Discussion

The analyses of the Gateshead EHCH vanguard were conducted using ITS segmented regression. ITS is regarded as the strongest quasi-experimental approach for evaluating longitudinal effects of interventions [11]. It allows the researcher to identify both an immediate impact of an intervention as well as the longer-term effect through changes in trends over time [12].

The findings of this study suggests that use of a larger data set with increased data points both before and after the introduction of the intervention resulted in differences in estimates in both the underlying time trends pre-vanguard and estimates of impact post vanguard with better fitting models and narrower confidence intervals. As a consequence, estimates of resource utilisation and associated costs differed between the two sets of analyses. In particular, the short-term model reported an overall increase in costs when using both a single tariff and a tariff per bed day. However, the long terms model found a large reduction in net costs using a single tariff relative to the short-term model and cost savings when using a tariff per day. Our findings regarding reductions in secondary care resource utilisation are consistent with findings from other evidence regarding the impact of EHCH [13]. However, one other study found little evidence of impact of the EHCH but this study had a markedly reduced follow-up period [14].

Natural experiments are useful for evaluating the impact of policy interventions such as the vanguards when routinely collected data are available for multiple time points both before and after the intervention occurs. As such, interrupted time-series designs offer a robust quasi-experimental alternative for evaluating effects of treatments or policies [15, 16]. In particular, in an interrupted time series (ITS) design, it is possible to detect whether the intervention has had an effect significantly greater than the underlying secular trend and they can often be performed inexpensively with the use of routinely collected data. Despite the popularity of ITS designs, there is a paucity of evidence regarding methodological standards and guidance regarding its use [17]. In particular, a common shortcoming of many studies is that they are underpowered [18]. Differing rules of thumb regarding number of time points before and after the intervention that are needed have been previously reported [12, 18, 19] ranging from eight to greater than ten. Despite this, there is a general consensus that the more data points there are both before and after the intervention, the better fit of the models i.e. narrower confidence intervals, standard errors are decreased, power is increased and hence detection of autocorrelation is more likely [18].

Rapid evaluations of specific policies using routinely collected data using natural experimental designs such as ITS or Difference in differences are problematic in that there are often too few data points post intervention to rapidly estimate impact in a reliable way. Policy makers and researchers must make sure that studies are adequately powered in order that policies are not based in inappropriate evidence. The short-term evaluation provided no evidence of return on investment for the ECHC vanguard in Gateshead. Had the policy making community relied on these results, it is unlikely that the model would have been scaled up and adopted wider. In contrast, the long-term evaluation was more encouraging with regard to a return on investment.

This issue of rapid evaluations with shorter longitudinal time frames for data points is further compounded with the introduction of complex interventions such as the EHCH vanguard. Complex interventions such as the EHCH require time to embed and take hold, with a minimum of 3 years suggested by Petch [20]. Additionally as previously reported [21], complex systems may require longer time frames before changes occur e.g. through a phase transition where there are long periods with little change in outcomes then large sudden changes in these outcomes occur.

Further suggesting the need to conduct longer-term evaluations rather than relying on short-term evaluations. In the case of EHCH vanguard, this complexity may be due to the need to continue to develop and maintain close working relationships, improving communication, teamwork and knowledge sharing, between different care-providers (e.g. Care home carers, GPs, nurses, and hospital specialists). Hence, we argue that evaluations of complex interventions using ITS may require more data points than the current literature suggests. When estimating impact of complex interventions, extra regard should be given to longer time periods in order to accurately estimate the full impact of such interventions.

### Limitations

One limitation of this study is the use of the post-code proxy of individuals over the age of 80 to identify care home residents. The proxy was required due to the lack of indicator within the routine data sets used that identifies whether an event (i.e. non-elective admission) is from a care-home resident. However, the use of this proxy may have resulted in an over-estimation of the resource use of care home residents. For example, an individual over the age of 80 living next door to a care home who required A&E admissions would be classified as a care home resident using this proxy.

Furthermore, it is acknowledged that controlled interrupted time series (CITS) would have offered a stronger study design as it combines ITS with one or more controls series, allowing both within and between group comparisons; strengthening control for potential confounders [22] .

Although The Kings Fund [23] recommend evaluations measure the effect of quality of care this economic evaluation of the EHCH vanguard was limited to analysing routinely collected data on secondary care usage. The impact of the EHCH on both primary care and the quality of care was not evaluated due to the unavailability of such data. However, future evaluations should attempt to measure the impact of the EHCH on the whole health and social care service as well as the quality of care.

The cost of the EHCH vanguard may be potentially over-estimated as it is assumed that practitioners that are part of the MDT are present for all 52 meetings each year. Additionally, it is assumed a care plan is made for all 1503 care home beds and is conducted twice a year for all beds. This is likely to be an overestimate as the care homes may not be filled to capacity at all times with all care.

## Conclusion

Although it is acknowledged that there is often a need for rapid evaluations in order to identify “quick wins” and to expedite learning within health and social care systems, we conclude that this may not be appropriate for natural experimental designs such as ITS for complex interventions. Our analyses suggests that care must be taken when conducting and interpreting the results of short-term evaluations using ITS methods, as they may produce misleading results and may lead to a misallocation of resources.

## Supplementary information

**Additional file 1.** PP (probability-probability) plots of normality.

**Additional file 2.** Tests for heteroscedasticity and autocorrelation A&E attendances.

## Data Availability

The data that support the findings of this study may be available from The North of England Commissioning Support Unit (NECS), but restrictions apply to the availability of these data, which were used under license for the current study, and are not publicly available.

## Abbreviations

A&E: Accident and emergency
EHCH: Enhanced Health in Care Homes
ITS: Interrupted time series
MDT: Multi-disciplinary Team
