# Supplementary material for: Rapid evaluation for health and social care innovations: challenges for “quick wins” using interrupted time series
Source: BMC Health Serv Res. 2019 Dec 13;19:964. doi: 10.1186/s12913-019-4821-7 (PMC6911271; doi:10.1186/s12913-019-4821-7)
Supplement: Supplementary file 1 — Additional file 1. PP (probability-probability) plots of normality. [file 12913_2019_4821_MOESM1_ESM.docx]

Additional **file 1:** PP (probability-probability) plots of Normality

A&E attendances

Short term model

Long term model

**Non-elective attendances**

Short term model

Long term model

**Outpatient appointments**

Short term model

Long term model

**Bed Days**

Short term model

Long term model
