# Supplementary material for: Rapid evaluation for health and social care innovations: challenges for “quick wins” using interrupted time series
Source: BMC Health Serv Res. 2019 Dec 13;19:964. doi: 10.1186/s12913-019-4821-7 (PMC6911271; doi:10.1186/s12913-019-4821-7)
Supplement: Supplementary file 2 — Additional file 2. Tests for heteroscedasticity and autocorrelation A&E attendances. [file 12913_2019_4821_MOESM2_ESM.docx]

**Additional file 2: Tests for Heteroscedasticity and Autocorrelation**

**A&E attendances**

Short term model

Long term model

**Non-elective attendances**

Short term model

Long term model

**Outpatient appointments**

Short term model

Long term model

**Bed Days**

Short term model

Long term model
